# Influence of chylomicron remnants on human monocyte activation *in vitro*

**DOI:** 10.1016/j.numecd.2010.02.019

**Published:** 2011-11

**Authors:** C. Bentley, N. Hathaway, J. Widdows, F. Bejta, C. De Pascale, M. Avella, C.P.D. Wheeler-Jones, K.M. Botham, C. Lawson

**Affiliations:** Cardiovascular Biology and Inflammation Research Group, Veterinary Basic Sciences, Royal Veterinary College, Royal College Street, London NW1 0TU, UK

**Keywords:** Monocytes/macrophages, Lipid mediators, Chemokines, Inflammation, Dietary fat

## Abstract

**Background and aims:**

Atherosclerosis is known to be an inflammatory disease and there is increasing evidence that chylomicron remnants (CMR), the lipoproteins which carry dietary fats in the blood, cause macrophage foam cell formation and inflammation. In early atherosclerosis the frequency of activated monocytes in the peripheral circulation is increased, and clearance of CMR from blood may be delayed, however, whether CMR contribute directly to monocyte activation and subsequent egress into the arterial wall has not been established. Here, the contribution of CMR to activation of monocyte pro-inflammatory pathways was assessed using an *in vitro* model.

**Methods and results:**

Primary human monocytes and CMR-like particles (CRLP) were used to measure several endpoints of monocyte activation. Treatment with CRLP caused rapid and prolonged generation of reactive oxygen species by monocytes. The pro-inflammatory chemokines MCP-1 and IL-8 were secreted in nanogram quantities by the cells in the absence of CRLP. IL-8 secretion was transiently increased after CRLP treatment, and CRLP maintained secretion in the presence of pharmacological inhibitors of IL-8 production. In contrast, exposure to CRLP significantly reduced MCP-1 secretion. Chemotaxis towards MCP-1 was increased in monocytes pre-exposed to CRLP and was reversed by addition of exogenous MCP-1.

**Conclusion:**

Our findings indicate that CRLP activate human monocytes and augment their migration *in vitro* by reducing cellular MCP-1 expression. Our data support the current hypothesis that CMR contribute to the inflammatory milieu of the arterial wall in early atherosclerosis, and suggest that this may reflect direct interaction with circulating blood monocytes.

## Introduction

Monocyte activation, triggering their adhesion to the endothelium and subsequent migration into the arterial intima, is an early event in atherogenesis [1–4]. Transformation into lipid-engorged macrophage foam cells follows, and leads to the appearance of fatty streaks, the first visible lesions in the vessel wall. Uptake of oxLDL by monocyte/macrophages is known to play a significant role in atherogenesis by stimulation of the secretion of pro-inflammatory cytokines, chemokines and other factors [5], but there is now considerable evidence to indicate that chylomicron remnants (CMR), the lipoproteins which transport fat of dietary origin from the gut to the liver, are also strongly atherogenic [6].

Lipids from food are absorbed in the gut and secreted into lymph in large, triacylglycerol (TG)-rich lipoproteins called chylomicrons which then pass into the blood via the thoracic duct. Here they undergo rapid lipolysis, a process that removes some of their TG and forms the smaller CMR which deliver the remaining TG, cholesterol and other lipids to the liver [7]. Chylomicron remnants are taken up and retained in the artery wall [8,9], and remnant-like particles have been isolated from the neointima of human atherosclerotic plaque and in animal models of atherosclerosis [10,11]. Delayed clearance of CMR correlates with the development of atherosclerotic lesions, and is associated with consumption of Western diets, obesity and type 2 diabetes [12,13].

Data from this laboratory and others has demonstrated that CMR are taken up by human macrophages derived from the human monocyte cell line THP-1 or from macrophages derived from freshly isolated monocytes [14,15] inducing foam cell formation [16], expression of genes involved in lipid metabolism [17] and modulation of pro-inflammatory cytokine expression [18,19]. Furthermore, CMR inhibit endothelium-dependent relaxation of isolated arteries [8,20,21], and trigger pro-inflammatory signal transduction in human endothelial cells (EC; [22]).

Monocytes are the precursors of macrophage foam cells and thus have a crucial role in atherogenesis. Under inflammatory conditions, activation of both monocytes and EC triggers expression of adhesion molecules, cytokines and vasoactive mediators and promotes monocyte adhesion to the endothelium and subsequent migration into the arterial wall [1,2,4]. The potential role of dietary fats in pro-inflammatory activation of circulating monocytes has not been explored experimentally, but TG-mediated expression of CD11b/Mac-1 has been reported after oral fat loading in normal healthy human volunteers [23,24].

Oxidative burst or reactive oxygen species (ROS) formation is a hallmark of monocyte activation and uptake of oxLDL by monocytes or monocyte-derived macrophages is known to be accompanied by ROS production [25]. We have shown previously that CMR induce ROS in THP-1 monocytes [26]. However, whether uptake of CMR by primary monocytes can induce ROS has not been investigated.

The aim of this study was to determine whether pro-inflammatory pathways are activated after monocyte interaction with CMR *in vitro* using primary human monocytes and model chylomicron remnant-like particles (CRLP). The effects of CRLP on; lipid accumulation; ROS generation; the secretion of the pro-inflammatory chemokines monocyte chemoattractant protein-1 (MCP-1) (also known as CCL2 in humans) and interleukin-8 (IL-8); and chemotaxis to MCP-1 by the cells were investigated. In addition, pharmacological inhibitors were used to gain information about the signalling pathways involved in the effects of CRLP on ROS generation and chemokine secretion.

## Methods

All chemicals and tissue culture reagents were from Sigma (Poole, Dorset, UK) unless otherwise stated. Tissue culture plastics were from Falcon Discovery Labware range (Fisher Scientific, UK), apart from Transwells which were from Greiner BioOne (Gloucestershire, UK). Pyrollidine dithiocarbamate (PDTC), U0126, apocynin, diphenyleneiodonium chloride (DPI), phenylarsine oxide (PAO) allopurinol and N-acetyl cysteine were all purchased from Sigma. U0124 was from Tocris Bioscience (Bristol, UK).

### Preparation of CRLP

CRLP were prepared by sonication of a lipid mixture containing 70% trilinolein, 2% cholesterol, 3% cholesteryl ester and 25% phospholipids in 0.9% NaCl (w/v) in Tricine Buffer (20 mM, pH7.4), followed by ultracentrifugation on a stepwise density gradient as described previously [27]. For apoE binding, lipid particles collected from the top layer of the final centrifugation step were incubated with the dialysed (18 h, 4 °C) d 1.063–1.21 g/ml fraction of human plasma (National Blood Transfusion Service, North London Centre, UK) as before [14]. CRLP containing apoE were then isolated by ultracentrifugation at d 1.006 g/ml (120,000 × *g*, 12 h, 4 °C), collected from the top layer, purified by a second centrifugation at the same density (202,000 × *g*, 4 h, 4 °C) and stored at 4 °C under argon until required [14,17]. All preparations were used within one week. To control for the possible presence of factors originating from plasma which may be present in the top layer after centrifugation, incubations with control preparations obtained by a similar procedure to that described for CRLP, but in the absence of the lipid particles, were included in all experiments. In all cases the data obtained with monocytes incubated with control preparations were not significantly different from those derived from cells incubated in medium alone.

### Isolation of human peripheral blood monocytes

Blood was taken by venepuncture from healthy volunteers into 15% EDTA tubes, with approval from the East London Research Ethics Committee. Monocytes were isolated by negative selection using RosetteSep according to the manufacturer's instructions (StemCell Technologies, London, UK). Cells were resuspended in RPMI containing 2 mM l-glutamine, 10,000 units/ml Penicillin, 10 mg/ml streptomycin and 10%(v/v) fetal bovine serum (PAA, Somerset, UK). Monocyte preparations were routinely stained with anti-CD14 antibody (Becton Dickinson, Oxford, UK) followed by flow cytometric analysis to verify purity.

### Oil Red O staining

1 × 10^6^ monocytes were incubated with CRLP (30 μg cholesterol/ml) (or a similar volume of control preparation), and incubated at 37 °C for 24 h. Cells were adhered to microscope slides by cytospin (Shandon, ThermoFisher Basingstoke, UK), and stained with Oil Red O as described previously [14]. Images were captured using a microscope mounted Canon digital camera and the extent of staining analysed Image J analysis software (NIH).

### Measurement of reactive oxygen species (ROS)

Monocytes were loaded with dihydrorhodamine-1,2,3 (final concentration 100 μM) for 10 min at room temperature and seeded onto white opaque 96 well tissue culture plates (2.5 × 10^4^ labelled monocytes/well). Pharmacological inhibitors were added for 10 min at 37 °C prior to addition of CRLP (7.5–30 μg/ml cholesterol) or a similar volume of control preparation. Plates were incubated at 37 °C for up to 24 h in 5% CO_2_ and fluorescence was measured, using a Wallac1410 fluorescent microtitre plate reader (Perkin Elmer, Beaconsfield, UK).

### Measurement of chemokine secretion

Monocytes were seeded at 5 × 10^5^ cells/well in 24 well tissue culture plates and CRLP (30 μg/ml cholesterol) or a similar volume of control preparation was added. Cells were exposed to pharmacological inhibitors for 10 min at 37 °C before addition of CRLP. After incubation at 37 °C for 6 or 24 h, cells were pelleted and the supernatants collected, snap frozen and stored at −80 °C until analysis using ELISA Duoset assay kits according to the manufacturer's instructions (R&D Systems, Oxford, UK).

### Chemotaxis assay

Monocytes were seeded at in 24 well tissue culture plates (5 × 10^5^ cells/well) and exposed to CRLP (30 μg cholesterol/ml) or a similar volume of control preparation for 24 h, then transferred to the upper chamber of Transwell plates in conditioned medium. RPMI supplemented with 10% FBS (600 μl) was placed in the lower Transwell chambers and recombinant human MCP-1 (CCL2) (10 ng/ml; R&D Systems) was added to lower and/or upper chambers. The plates were incubated for 4 h and the number of cells that had migrated into the lower chamber after this time were counted by flow cytometry (Beckman Coulter, Oxford UK).

### Statistical analysis

Two way ANOVA followed by Bonferroni's multiple comparison test was used to analyse ROS production and the effects of pharmacological inhibitors on cytokine production, and one way ANOVA followed by the Tukey Kramer multiple comparison test was used for all other data, except where indicated otherwise.

## Results

### Induction of lipid accumulation in monocytes

**Figure 1:**
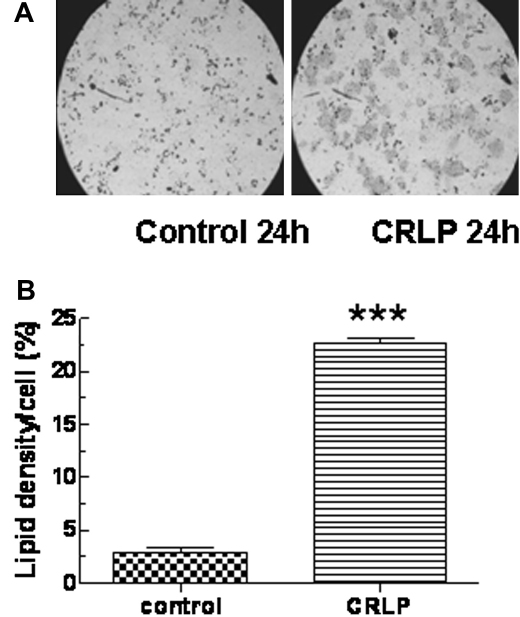
Lipid accumulation in CRLP-treated human monocytes. Primary human monocytes were incubated with control preparations (see Methods) or CRLP (final concentration 30 μg/ml cholesterol) for 24 h. (A) Monocytes were adhered to microscope slides by cytospin and stained with Oil Red O. (B) Digital image analysis was carried out to determine lipid uptake. Data are given as mean ± sem of five experiments using monocytes from five individual donors. ***p < 0.001 CRLP-treated vs untreated or control treated monocytes.

Incubation of isolated monocytes with CRLP for 24 h resulted in increased intracellular accumulation of lipid as assessed by Oil Red O staining (Figure 1A). Quantification of the staining density using digital image analysis showed that there was a highly significant increase in the lipid content of CRLP-treated monocytes when compared to cells exposed to the control preparation (*P* < 0.001, paired Student's *t* test) (Figure 1B).

### Generation of reactive oxygen species

**Figure 2:**
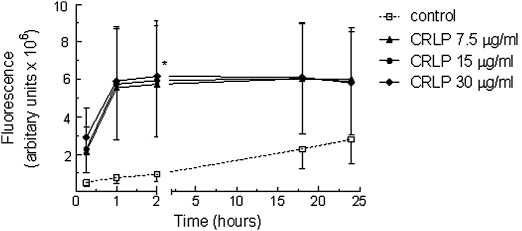
ROS generation by monocytes incubated with CRLP for up to 48 h. Freshly isolated monocytes were pre-loaded with dihydrorhodamine-1,2,3 before addition varying concentrations of CRLP (0–30 μg cholesterol/ml) or an equal volume of control preparation, and fluorescence was detected using a microtitre plate reader at times up to 24 h. Data are as the mean ± sem of four experiments using monocytes from four individual donors *p < 0.05 CRLP (all concentrations) compared to control treated cells.

**Figure 3:**
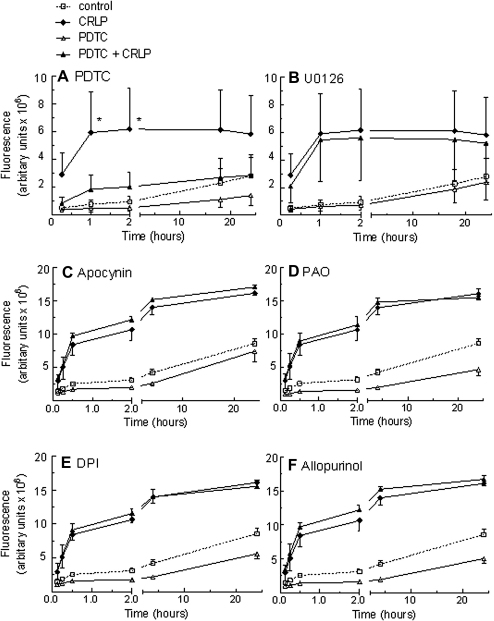
Inhibition of CMR-mediated ROS generation. Freshly isolated monocytes were loaded with DHR 1,2,3 then pre-treated with 20 μM PDTC (A), 1 μM MEK inhibitor U0126 (B) 100 μM Apocynin (C), 100 μM NADPH oxidase inhibitor PAO (D), 10 μM NADPH oxidase inhibitor DPI (E), 100 μM xanthine oxidase inhibitor allopurinol (F), before addition of CRLP (30 μg cholesterol/ml) or control preparations (see Methods), and fluorescence was detected using a microtitre plate reader at increasing times. Plots are all mean ± sem of four experiments using monocytes from four individual donors *p < 0.05 CRLP compared to CRLP + inhibitor treated cells.

On incubation of dihydrorhodamine-1,2,3-loaded monocytes with CRLP (7.5–30 μg cholesterol/ml) there was a rapid increase in ROS formation in comparison to that observed in control cells; after 1 h exposure to CRLP at a dose of 7.5 μg cholesterol/ml there was a 7.5 fold increase which was maintained for at least 24 h and was not dose dependent (Figure 2). PDTC, a well-characterised antioxidant with reported ability to inhibit NF-κB activity, reduced both basal and CRLP-induced ROS production (Figure 3A). In contrast, inhibitors of NADPH oxidase (apocynin; PAO; DPI Figure 3C–E) or xanthine oxidase (allopurinol; Figure 3F) had no significant effect on ROS production in CRLP-treated cells. Similarly, neither the MEK inhibitor U0126 (Figure 3B) nor its inactive analogue U0124 (data not shown), affected ROS generation in the presence of CRLP.

### Modulation of chemokine secretion

**Figure 4:**
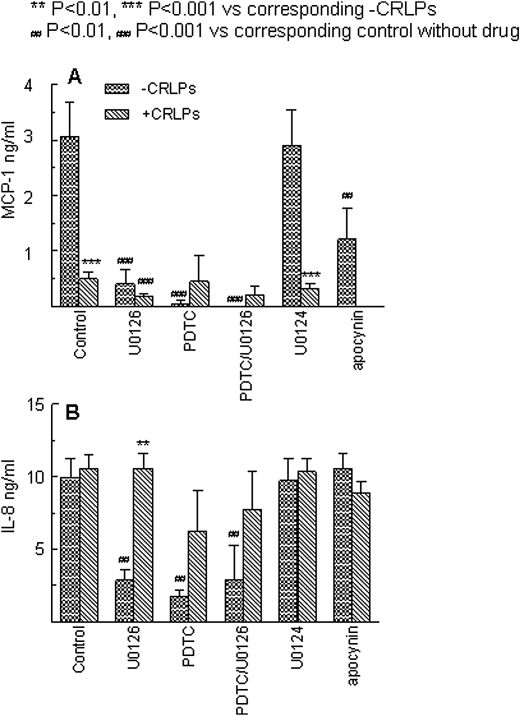
Effect of the inhibitors U0126, PDTC, and apocynin on chemokine secretion by primary human monocytes. Freshly isolated monocytes were pre-incubated without (control) or with U0126 (1 μM) PDTC (100 μM) or apocynin (100 μM) for 10 min before addition of CRLP (30 μg/ml cholesterol). Supernatant was collected after 20 h and levels of MCP-1 (A) or IL-8 (B) were measured by ELISA. Data are given as mean ± sem of five experiments using monocytes from five individual donors *p < 0.05; **p < 0.01; ***p < 0.001 compared to control treated cells.

Freshly isolated human monocytes were incubated with or without CRLP for 6 or 24 h and the secretion of MCP-1 and IL-8 into the medium was measured (*n* = 5). In the absence of CRLP, the cells secreted high quantities of MCP-1 (CCL2) (5.01 ± 1.58 ng/ml) and IL-8 (CXCL8) (1.54 ± 0.24 ng/ml) after 24 h. Secretion of MCP-1 was decreased by CRLP treatment and this effect was significant after 24 h (6 h, 2.78 ± 0.84 ng/ml; 24 h, 0.65 ± 0.01 ng/ml (*P* < 0.05)) (Figure 4A), whilst IL-8 secretion into the medium was increased at 6 h (3.34 ± 0.30 ng/ml, *P* < 0.001) and returned to control levels by 24 h (2.77 ± 0.11 ng/ml) (Figure 4B).

Constitutive secretion of both MCP-1 and IL-8 was significantly reduced by treatment with U0126. Production of MCP-1 and IL-8 was also inhibited by PDTC, whereas the NADPH oxidase inhibitor, apocynin, had no effect (Figure 4A, B). Incubation with CRLP did not influence the reduced MCP-1 secretion observed following treatment with U0126 or PDTC (Figure 4A) but restored IL-8 secretion to constitutive levels in the presence of either inhibitor (Figure 4B.).

### Monocyte chemotaxis

**Figure 5:**
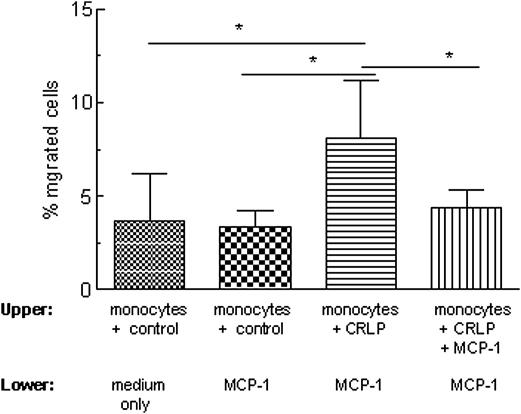
Monocyte chemotaxis towards MCP-1 after pre-exposure to CRLP for 24 h. Monocytes were pre-incubated with CRLP (30 μg/ml cholesterol) or control preparations (see Methods), for 24 h before being placed into transwells (0.6 × 10^6^ cells/well) without washing. The lower chamber was flooded with media with or without 10 ng/ml MCP-1 and incubation was continued for a further 4 h before counting cells that had migrated to the lower chamber by flow cytometry. Data are given as mean ± sem of six experiments using monocytes from six individual donors. *p < 0.05.

We hypothesised that the CRLP-driven reduction in MCP-1 secretion may result in increased monocyte chemotaxis due to the resulting increased MCP-1 concentration gradient in the monocyte microenvironment. This was investigated *in vitro* by testing the migration of cells towards MCP-1 using Transwell chambers (Figure 5). After pre-exposure to control preparations for 24 h, the number of monocytes migrating to the lower chamber of the Transwells was not significantly different in the presence or absence of MCP-1 in the lower chamber (Figure 5). Pre-treatment with CRLP, however, caused a significantly higher percentage of monocytes to migrate towards recombinant MCP-1. Addition of recombinant MCP-1 to CRLP-treated monocytes before commencement of the migration assay abolished this effect (Figure 5).

## Discussion

Recent studies have suggested that the interaction of CMR with monocytes may play a part in their atherogenic effects [22,24,27]. Studies in human leukocytes isolated from subjects after ingestion of a fat meal have shown that they take up TG-rich lipoproteins obtained from the same subjects 4 h postprandially, and that this results in increased expression of activation markers including CD11b [23,24]. This is likely to be significant for development of atherosclerosis, particularly when the removal of CMR from the blood is delayed as occurs in relatively common conditions such as obesity and type 2 diabetes [28]. Chylomicron remnants have been shown to influence cytokine and chemokine expression in monocyte-derived THP-1 macrophages [18,19], however, the potential activation of pro-inflammatory, pro-atherogenic signalling in primary human undifferentiated monocytes by CMR has not been explored previously. In the present study we have shown that CRLP cause lipid accumulation in primary human monocytes and that this is associated with rapid and prolonged ROS production, the modulation of secretion of the chemokines IL-8 and MCP-1 and increased chemotaxis towards MCP-1.

Since CMR uncontaminated with other TG-rich lipoproteins such as chylomicrons and very lowdensity lipoprotein (VLDL) cannot be obtained easily from human blood, we used model CRLPs containing human apoE for our experiments. In extensive previous work, we and others have shown that these particles mimic the effects of physiological CMR both *in vivo* and *in vitro* [7,14,29].

Previous work by Alipour et al. [23] suggested that leukocytes isolated postprandially from volunteers fed a high fat diet take up lipid from TG-rich lipoprotein such as CMR, since they became enriched in meal-derived fatty acids. Our experiments, however, demonstrate directly that exposure of human monocytes to CRLP causes lipid to accumulate inside the cells (Figure 1), and thus provide the first direct evidence of CMR uptake by monocytes.

Oxidative or respiratory bursts in monocytes generate reactive oxygen species (ROS) primarily as a defence mechanism against infection, but are also generated by these cells during other inflammatory reactions. In the current study, CRLP were found to cause a large (x 7.5–8), rapid and prolonged increase in the generation of ROS in monocytes (Figure 2). Previous studies have shown that human monocytes generate ROS in response to oxidised LDL [25], and CMR from rats have been found to upregulate ROS production by the THP-1 human monocyte cell line [30]. However, this is the first report to demonstrate that CRLP promote ROS production in primary human monocytes.

The ERK1/2 and NF-κB pathways have been implicated in inflammation-driven ROS generation and cardiovascular disease [4,31]. U0126 is a well defined pharmacological inhibitor of MEK, the direct upstream regulator of ERK1/2, and PDTC is often used to block NF-κB activation, since it stabilizes the cytosolic NF-κB inhibitor, IκB-α, via inhibition of IκB-α ubiquitination [32,33] and alters the oxidation state of NF-κB subunits [34]. We used these inhibitors, therefore, to determine the role of the ERK1/2 and NF-κB pathways in CRLP-induced ROS production in human monocytes. U0126 was shown to prevent the accumulation of ROS in untreated cells, but did not affect CRLP-mediated ROS generation. In contrast, PDTC inhibited ROS production in both control and CRLP-treated cells (Figure 3A). These results are consistent with the previous finding that ingestion of a meal high in butter or walnut oil fat activates NF-κB in peripheral blood mononuclear cells from healthy volunteers [35] and suggest that the induction of ROS generation by CMR in human monocytes is mediated by NF-κB, but that the ERK1/2 pathway is not involved. Interestingly, in a recent study from our group we showed that CRLP downregulate NF-κB activity in macrophages derived from THP-1 monocytes [18] suggesting that there are differences in the effects of CRLP on monocytes as compared to macrophages.

NADPH oxidase acts as a catalyst of the transfer of electrons from NADPH to O_2_, which results in the formation of superoxide anion and other ROS involved in microbial defence [36]. More recently, NADPH oxidase has been shown to be a family of enzymes critically involved in the tissue damage caused by oxidative stress in atherogenesis [37]. TNF-induced ROS production has been reported to occur through NF-κB-mediated transcriptional regulation of the NADPH oxidase genes in MonoMac1, a human monocyte cell line [38]. Thus, we sought to determine the role of NADPH oxidases in CRLP-stimulated ROS production using the NADPH oxidase inhibitors apocynin, DPI and PAO [39–41]. However, none of the inhibitors affected the prolonged CRLP-mediated generation of ROS. Likewise, allopurinol, an inhibitor of xanthine oxidase, which has also been implicated in ROS generation in atherosclerosis [42], did not prevent the increase in ROS found in monocytes in response to CRLP. We conclude, therefore, that CRLP do not stimulate ROS production via modification of either NADPH oxidase or xanthine oxidase activity.

It is well established that human peripheral blood monocytes secrete MCP-1 and IL-8 and that synthesis of these chemokines increases following exposure to pro-inflammatory stimuli. A surprising finding of the current study, therefore, is that CRLP cause a marked decrease in monocyte MCP-1 secretion in monocytes, particularly since previous studies have shown that both CMR and ROS production induce MCP-1 secretion from vascular smooth muscle cells [43], and that agents that reduce ROS formation suppress NF-κB dependent MCP-1 secretion in monocytes *in vitro* [44]. In contrast, IL-8 secretion by the monocytes was transiently increased after 6 h incubation with CRLP. However, since CRLP reversed the inhibition caused by PDTC or U0126 (Figure 4B), we conclude that their stimulatory effect is not mediated via the MEK/ERK pathway.

Considering the potential effects of the decrease in MCP-1 secretion caused by CMR on the migration of monocytes, we hypothesised that reduced levels of monocyte-derived MCP-1 in the microenvironment of the blood vessel lumen may increase the MCP-1 chemotactic gradient across the endothelium. In order to test this, we investigated how CRLP pre-treatment affected monocyte chemotaxis across a transwell filter towards MCP-1. As predicted, decreased MCP-1 levels in the culture medium of monocytes after treatment with CRLP enhanced the subsequent migration of the cells towards a higher concentration of MCP-1, and furthermore, this effect was reversed by addition of exogenous MCP-1 to the culture medium after the incubation with CRLP (Figure 5). We propose, therefore that CMR have an overall pro-migratory effect on circulating monocytes via down-regulation of their constitutive MCP-1 secretion (Figure 4A). Enhancement of IL-8 secretion by CMR may also increase monocyte migration, since this chemokine has recently been reported to activate monocytes during firm adhesion to the endothelium [45].

In summary, this study demonstrates that CRLP cause lipid accumulation in peripheral blood monocytes and induce prolonged ROS production. Moreover, CRLP inhibit MCP-1 secretion and enhance their migration towards MCP-1. These findings indicate a pro-inflammatory, pro-migratory effect of CMR on peripheral blood monocytes, and support the current hypothesis that CMR contribute to the inflammatory milieu seen in susceptible areas of the artery wall in early atherosclerosis.
